# Parotid gland dose reduction in the hippocampus avoidance whole-brain radiotherapy using helical tomotherapy

**DOI:** 10.1093/jrr/rrab107

**Published:** 2021-11-27

**Authors:** Hidetoshi Shimizu, Koji Sasaki, Takahiro Aoyama, Hiroyuki Tachibana, Yutaro Koide, Tohru Iwata, Tomoki Kitagawa, Takeshi Kodaira

**Keywords:** brain metastases (BM), helical tomotherapy (HT), hippocampus avoidance with whole-brain radiotherapy (HA-WBRT), parotid gland dose, radiotherapy

## Abstract

The present study aimed to reduce the parotid gland dose in the hippocampus avoidance with whole-brain radiotherapy (HA-WBRT) using the helical tomotherapy (HT). Ten patients who had previously undergone WBRT were randomly selected and enrolled in this study. During the treatment planning, two different techniques to the jaw were applied for each patient, namely, 1.0 cm fixed jaw and 2.5 cm dynamic jaw. To efficiently reduce the dose in the bilateral parotid glands, directional block (DB) mode was set. The DB is a function of a treatment planning system for the dose reduction in organs at risk. The standard HA-WBRT plan which did not reduce the parotid gland dose was also designed to compare the plan quality. Compared with the standard HA-WBRT plan, the parotid gland dose could be reduced by approximately 70% without extending the delivery time by adding the parotid gland on the DB mode to the dose constraint. In addition, the differences in dosimetric parameters observed between the plans employing the 1.0 cm fixed jaw and 2.5 cm dynamic jaw were almost negligible. Moreover, delivery time in the 2.5 cm dynamic jaw could be greatly reduced by 60% compared with that in the 1.0 cm fixed jaw.

## INTRODUCTION

Brain metastases (BM) occur in approximately 20% of the patients with cancer [1]. Historically, the prognosis of patients with BM has been poor; however, it has become evident that not all patients with BM have the same poor prognosis. Therefore, the use of an identical management strategy for all patients is no longer appropriate [2]. In this context, this work focuses on whole-brain radiotherapy (WBRT), which is an important technique for the treatment of patients with multiple BM.

In WBRT, there are two notable risk organs in addition to the lens and the eye, namely, the hippocampus and parotid glands. The hippocampus suffers cognitive damage and deterioration caused by irradiation [3]. This fact might delay and limit the use of WBRT in initial treatment for BM. Therefore, the hippocampus avoidance with WBRT (HA-WBRT), using intensity-modulated radiotherapy (IMRT), to prevent cognitive deterioration has been reported as a promising solution. In a single-arm phase II trial Radiation Therapy Oncology Group (RTOG) 0933 [4], HA-WBRT showed significant benefit in preserving the short-term memory function. Additionally, in phase III trial NRG Oncology CC001 [5], the use of the combination of HA-WBRT plus memantine was an excellent option to better preserve cognitive function and address patient-reported symptoms; however, there was no difference in intracranial progression-free survival and overall survival. Considering the other risk organ affected, i.e. the parotid glands [6–11], Noh *et al.* reported that 12 (37.5%) and one (3.1%) patients received the mean doses of >20 Gy or > 25 Gy in WBRT, respectively [6]. These results indicated that some patients exceeded dose tolerances of the parotid glands. Indeed, Deasy *et al.* demonstrated that the mean dose that prevents severe xerostomia in one parotid gland and both glands was 20 and 25 Gy, respectively [12]. To date, no studies have reported that the parotid gland dose in WBRT was associated with clinically significant xerostomia. However, Wang *et al.*, in a cohort study with 73 WBRT patients, revealed that xerostomia was statistically significantly associated with the parotid gland dose [11]. Thus, the parotid gland dose reduction is necessary to prevent xerostomia related with HA-WBRT.

This study focused on helical tomotherapy (HT) to reduce parotid gland dose. HT is a preferred delivery technique for HA-WBRT because it allows superior dose homogeneity for the target organs, allowing a low hippocampus dose compared with volumetric arc radiotherapy (VMAT) and step and shoot IMRT [13, 14]. The excellent dose distribution characteristic of HT possibly allows the use of lower parotid gland dose. However, there are no reports on parotid gland dose reduction in HA-WBRT using HT. This study aimed to reduce the parotid gland dose in HA-WBRT using HT.

## MATERIALS AND METHODS

### Patient selection

Ten patients who previously underwent WBRT were randomly selected and enrolled in this study. The investigated patients were immobilized from the top of the head to the chin with a thermoplastic mask, and 3 mm slice computed tomography (CT) images (Aquilion LB, Canon Medical Systems Co.) were acquired. In addition, 3-Tesla magnetic resonance imaging (MRI) (Signa HDxt, GE Healthcare) was performed to delineate the hippocampus. The slice thickness for the axial plane of the MRI image set was 3 mm. This study was approved by the institutional review board of our hospital (No. 2020–1-021).

### Delineation of target volumes and organs at risk

The CT-image sets were imported into the treatment planning system (RayStation, version 6.2.0, RaySearch Laboratories). The whole brain was delineated as the clinical target volume (CTV). A planning target volume (PTV) was created by adding 5 mm isotropic margins to the CTV. The high-risk organs delineated were the hippocampus, bilateral parotid glands and lens. The hippocampus was delineated according to an atlas [15], with reference to the T1-weighted MRI image. The planning organ at risk volume (PRV) of the hippocampus was defined by adding a 5 mm isotropic margin to the hippocampus. Finally, PTV_eval_ was defined as the volume obtained by subtracting the PRV of the hippocampus from the PTV to avoid dose competition in the overlap between the PTV and PRV of the hippocampus based on the RTOG 0933 protocol [4]. The ring-shaped volume of the 1.0 cm width was set at 1.0 cm outside of the entire PTV_eval_ to obtain better a dose conformity of PTV_eval_.

### Treatment plan

The delineation of targets and organs at risk was imported into the treatment planning system (Precision, version 2.0.1.1, Accuray, Sunnyvale, CA). The Radixact (Accuray, Sunnyvale, CA) was selected as the treatment machine. The modulation factor and pitch, which are the specific treatment planning parameters for Radixact, were set at 2.0 and 0.43, respectively. Two techniques (1.0 cm fixed jaw and 2.5 cm dynamic jaw) were applied to the jaw, the remaining treatment planning parameter, for each patient. The 1.0 cm fixed jaw was the technique in which a constant jaw size of 1.0 cm was used, whereas the 2.5 cm dynamic jaw was the technique that maintained a constant jaw width of 2.5 cm and dynamically adapted the jaw width only at the cranial and caudal edges of the target volume. The dose prescribed was set as 30 Gy to the 95% volume of PTV_eval_, being delivered in 10 fractions. To more efficiently reduce the dose in the bilateral parotid glands than normal dose optimization, they were set as a directional block (DB). The DB is a function of Precision for the dose reduction in organs at risk, not including beamlets that reach the parotid gland before passing through the target during dose optimization computing [16]. Dose optimization was applied to satisfy the following constraints: dose to 98% of the volume (D_98%_) ˃ 25 Gy and D_2%_ ˂ 37.5 Gy for the PTV_eval_; maximum dose ˂16 Gy for the hippocampus; maximum dose ˂8 Gy for the lens; and maximum dose ˂37.5 Gy for outside the PTV to avoid unexpectedly high dose to the eye and the optic nerve. The optimization parameters are listed in Table 1 and were standardly used for all patients, with 200 iterations conducted. The importance or penalty shows the weight of the optimization cost function, which applies for all dose constraints or one dose constraint in each structure, respectively. Owing to the specification of Precision, it is necessary to set dose optimization settings even for the parotid glands, which were set as DB. These dose optimization settings are included in the optimization cost function to control the beamlets that reach the parotid glands after passing via the target or the scattered dose to the parotid glands. The grid sizes (International Electrotechnical Commission [IEC]-X, Y and Z) of the final calculation were 1.07, 3.0 and 1.07 mm, respectively. A plan that did not add the parotid gland on the DB mode to the dose constraint (i.e. without the parotid gland dose reduction) was also designed through a similar dose optimization procedure to compare the plan quality.

**Table 1:** Dose optimization settings of the treatment planning

(a) Target							
Structure	Importance	Maximum dose [Gy]	Maximum dose penalty	DVH volume [%]	DVH dose [Gy]	Minimum dose [Gy]	Minimum dose penalty
PTV_eval_	1000	30	1000	95	30	30	1000
(b) Organs at risk and the ring-shaped volume							
Structure	Importance	Maximum dose [Gy]	Maximum dose penalty	DVH volume [%]	DVH dose [Gy]	DVH penalty	
Lens	100	5	200	1	4	200	
The PRV of the Hippocampus	200	15	500	100	7	1500	
Right parotid gland^*^	100	5	200	1	4	200	
Left parotid gland^*^	100	5	200	1	4	200	
The ring-shaped volume	1	30	1	70	10	100	
				30	15	100	
				5	25	100	

^*^Only for HA-WBRT plan with the parotid gland dose reduction by DB mode

PTV, planning target volume; DVH, dose volume histogram; PRV, planning organ at risk volume; HA-WBRT, hippocampus avoidance whole-brain radiotherapy

The use of ArcCHECK (Sun Nuclear Corporation) allowed to confirm that all the plans were clinically available with a passing rate of ≥90% on a global gamma criterion of dose difference of 3% and distance to agreement of 2 mm with a dose threshold of 10%.

### Data analysis

#### Comparison of the dosimetric parameters in the HA-WBRT plan with and without parotid gland dose reduction

To compare the dosimetric parameters in the HA-WBRT plan with and without parotid gland dose reduction, the following parameters were calculated: D_98%_; D_50%_; D_2%_; the volume receiving at least 30 Gy (V_30Gy_); homogeneity index (HI) [17] and conformity index (CI) [17] of the PTV_eval_; the maximum and mean doses for the lens and bilateral parotid glands; the D_100%_ and maximum dose for the hippocampus; and the D_100%_ for the body. The HI and CI values were calculated as described in the Appendix.

#### Comparison of the delivery time in the HA-WBRT plan with and without parotid gland dose reduction

To compare the delivery time in the HA-WBRT plan with and without parotid gland dose reduction, the delivery time was read out from the treatment planning report created by Precision.

### Statistical analysis

Freidman test was used to compare the difference in the dosimetric parameters or delivery time among the DB mode patterns (including or not including the parotid gland) and jaw patterns (1.0 cm fixed jaw and 2.5 cm dynamic jaw). In the presence of a significant difference detected using the Freidman test, Wilcoxon signed-rank test with Bonferroni’s correction was performed. All the statistical analyses were performed with EZR [18], which is a modified version of R commander designed to add statistical functions frequently used in the biostatistics. *P-*values of <0.05 were considered statistically significant.

## RESULTS

### Comparison of the dosimetric parameters in the HA-WBRT plan with and without parotid gland dose reduction

The values of the dosimetric parameters in the HA-WBRT plan with and without parotid gland dose reduction are listed in Table 2. Freidman test showed a significant difference in all dosimetric parameters (*p* < 0.05). When the jaw size was the same (comparison of [1] and [2] or [3] and [4] in Table 2), both the maximum and mean doses for bilateral parotid glands in the HA-WBRT plan with the DB mode decreased significantly (approximately 30%) compared with the values without the DB mode (*p* < 0.05, Wilcoxon signed-rank test with Bonferroni’s correction). Comparing the 1.0 cm fixed jaw and the 2.5 cm dynamic jaw plans with the parotid gland dose reduction by DB mode (comparison of [2] and [4] in Table 2), the 1.0 cm fixed jaw plan was superior except for the V_30Gy_ of PTV_eval_ to the 2.5 cm dynamic jaw plan (*p* < 0.05). However, the difference observed was clinically negligible.

**Table 2:** Dosimetric parameters and dose delivery time in the HA-WBRT plan (^*^: with the parotid gland dose reduction by DB mode)

Structure	Index	(1) 1.0 cm fixed Jaw	(2) 1.0 cm fixed Jaw^*^	(3) 2.5 cm dynamic jaw	(4) 2.5 cm dynamic jaw^*^	Freidman test *p* value	Wilcoxon signed-rank test with Bonferroni’s correction *p* value		
							(1) vs (2)	(3) vs (4)	(2) vs (4)
PTV_eval_	D_98%_ [Gy]	26.0 ± 0.6	26.1 ± 0.7	25.0 ± 0.2	25.1 ± 0.3	<0.05	<0.05	0.26	<0.05
	D_50%_ [Gy]	31.0 ± 0.3	31.0 ± 0.3	32.5 ± 0.7	32.5 ± 0.6	<0.05	1.00	1.00	<0.05
	D_2%_ [Gy]	31.8 ± 0.4	31.8 ± 0.3	33.8 ± 0.7	33.9 ± 0.6	<0.05	1.00	0.45	<0.05
	V_30Gy_ [%]	94.7 ± 0.3	94.7 ± 0.3	94.9 ± 0.0	94.9 ± 0.0	<0.05	1.00	1.00	0.21
	HI [%]	19.3 ± 3.2	19.1 ± 3.2	29.1 ± 3.0	29.4 ± 2.9	<0.05	0.06	1.00	<0.05
	CI	0.82 ± 0.02	0.82 ± 0.02	0.73 ± 0.03	0.73 ± 0.03	<0.05	0.16	<0.05	<0.05
Lens	Maximum dose [Gy]	5.1 ± 0.4 s	5.1 ± 0.4	5.6 ± 0.3	5.6 ± 0.3	<0.05	0.58	1.00	<0.05
	Mean dose [Gy]	3.8 ± 0.2	3.8 ± 0.2	4.1 ± 0.2	4.2 ± 0.2	<0.05	1.00	1.00	<0.05
Bilateral parotid glands	Maximum dose [Gy]	22.8 ± 2.5	7.2 ± 1.4	26.7 ± 2.4	9.4 ± 2.3	<0.05	<0.05	<0.05	<0.05
	Mean dose [Gy]	8.7 ± 1.6	2.5 ± 0.3	11.6 ± 2.1	2.7 ± 0.3	<0.05	<0.05	<0.05	<0.05
Hippocampus	D_100%_ [Gy]	8.6 ± 0.4	8.6 ± 0.4	10.2 ± 0.6	10.2 ± 0.5	<0.05	0.09	1.00	<0.05
	Maximum dose [Gy]	13.0 ± 0.8	13.0 ± 0.8	14.5 ± 1.0	14.5 ± 0.9	<0.05	0.18	1.00	<0.05
Body	V_30Gy_ [cc]	1783 ± 146	1778 ± 142	2017 ± 173	2007 ± 168	<0.05	0.22	0.06	<0.05
Dose delivery time [s]		836.6 ± 40.0	854.2 ± 43.0	362.6 ± 18.9	375.2 ± 20.0	<0.05	<0.05	<0.05	<0.05

HA-WBRT, hippocampus avoidance whole-brain radiotherapy; DB, directional block; PTV, planning target volume; HI, homogeneity index; CI, conformity index

**Fig. 1:**
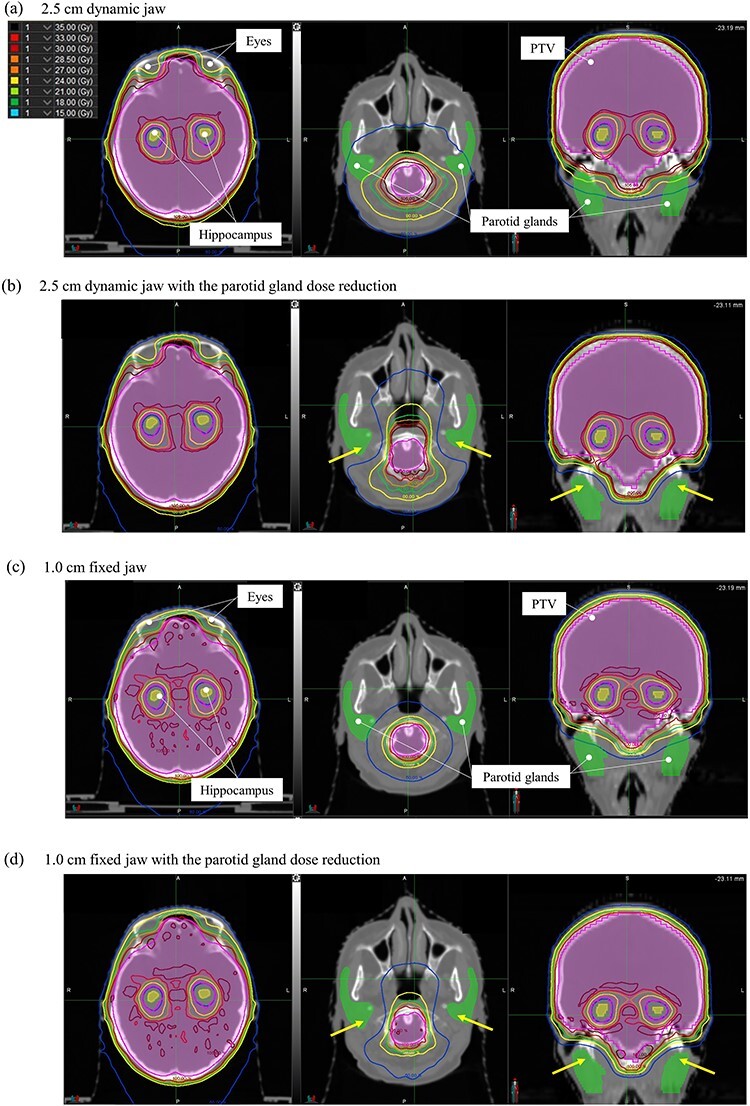
Dose distribution of the planes of the hippocampus and parotid gland in a representative case. (a)–(d) show the dose distributions of the HA-WBRT plans without the parotid gland dose reduction using the 2.5 cm dynamic jaw, with the DB mode using the 2.5 cm dynamic jaw, without the parotid gland dose reduction using the 1.0 cm fixed jaw and with the DB mode using the 1.0 cm fixed jaw, respectively. The yellow arrows show the area in which the parotid gland dose had decreased. HA-WBRT, hippocampus avoidance whole-brain radiotherapy.

**Fig. 2:**
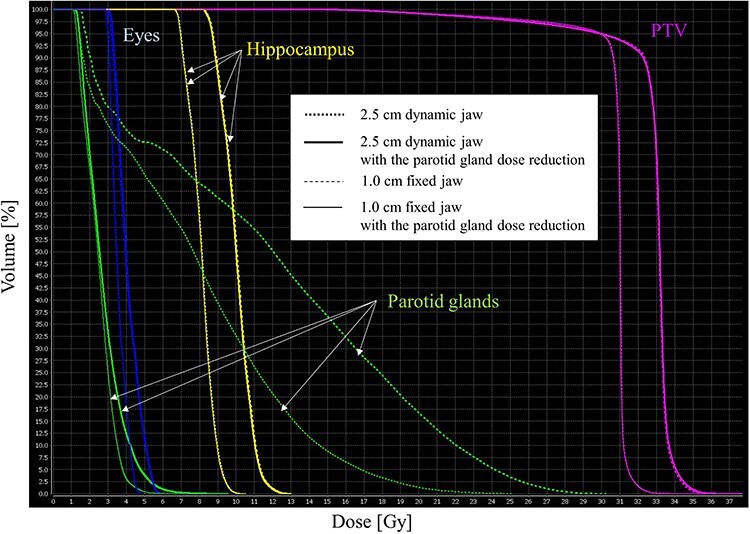
Dose volume histogram (DVH) of the same patient descibed in Fig. 1. Thick-dashed, thick-solid, thin-dashed and thin-solid lines show the DVH curves of the HA-WBRT plans without the parotid gland dose reduction using the 2.5 cm dynamic jaw, with the parotid gland dose reduction using the 2.5 cm dynamic jaw, without the parotid gland dose reduction using the 1.0 cm fixed jaw and with the parotid gland dose reduction using the 1.0 cm fixed jaw, respectively. Without depending on the jaw modes, the parotid gland dose was reduced by adding the parotid gland on the DB mode.

Fig. 1 shows the dose distribution of the hippocampus and parotid gland planes in a representative case. Fig. 1 depicts the dose distributions of the HA-WBRT plans without the parotid gland dose reduction using the 2.5 cm dynamic jaw (Fig. 1a), with the parotid gland dose reduction using the 2.5 cm dynamic jaw (Fig. 1b), without the parotid gland dose reduction using the 1.0 cm fixed jaw (Fig. 1c) and with the parotid gland dose reduction using the 1.0 cm fixed jaw (Fig. 1d). Fig. 2 shows the dose volume histogram of the same investigated case. In the same case, by adding the parotid gland on the DB mode to the dose constraint, the HA-WBRT plan could reduce the parotid gland dose while sparing the hippocampus and lens (the arrows in the [b] and [d] of Fig. 1). In addition, without depending on the jaw modes, the parotid gland dose greatly reduced by adding the parotid gland on the DB mode to the dose constraint.

### Comparison of the delivery time in the HA-WBRT plan with and without parotid gland dose reduction

Table 2 listed the delivery time in the HA-WBRT plan with and without parotid gland dose reduction. When the jaw mode was the same (comparison of [1] and [2] or [3] and [4] in Table 2), the delivery time showed a significant difference between the HA-WBRT plans with and without parotid gland dose reduction (*p < 0.05*, Wilcoxon signed-rank test with Bonferroni’s correction). Notwithstanding, the observed difference was extremely small, i.e. 10–20 s. Finally, the delivery time in the 2.5 cm dynamic jaw was significantly shortened (i.e. approximately 60%) compared with the 1.0 cm fixed jaw (*p* < 0.05).

## DISCUSSION

This study aimed to reduce the parotid gland dose in HA-WBRT using HT. In this study, it was demonstrated that the dose was reduced without an excessive increase in the delivery time by adding the parotid gland on the DB mode to the dose constraint. In addition, the difference observed in the dosimetric parameters between the plans employing the 1.0 cm fixed jaw and 2.5 cm dynamic jaw was extremely small, and the delivery time in the 2.5 cm dynamic jaw was 60% shortened compared with that in the 1.0 cm fixed jaw.

Sood *et al.* reported that VMAT could reduce not only the doses to the hippocampus but also to other healthy organs, including the parotid gland [19]. In the same study, the authors reported that the maximum and mean doses to both parotid glands were 15.7 ± 5.0 Gy and 4.4 ± 1.9 Gy, respectively [19]. Oh *et al.* demonstrated that VMAT with a head tilting device allowed to reduce the parotid gland dose as well as the hippocampus dose [20]. According to the report from Oh *et al.*, the maximum and mean doses to both parotid glands were 19.28 ± 5.19 Gy and 4.77 ± 1.97 Gy, respectively [20]. These studies used VMAT as the delivery technique. In this study, the parotid gland was added on the DB mode of HT to the dose constraint. The maximum and mean doses to both parotid glands in the 1.0 cm fixed jaw were 7.2 ± 1.4 Gy and 2.5 ± 0.3 Gy, respectively. In addition, in the 2.5 cm dynamic jaw, the maximum and mean doses were 9.4 ± 2.3 Gy and 2.7 ± 0.3 Gy, respectively. Although in the study conducted by Oh *et al*. the authors did not intend dose reduction in the parotid gland, the values obtained in this study were approximately half compared with the values reported by Sood *et al*. [19] and Oh *et al.* [20], being evaluated to be obviously lower. Deasy *et al.* reported that the mean dose to each parotid gland should be kept as low as possible because the lower mean dose to the parotid gland results in better function, even for relatively low mean doses (<10 Gy) [12]. We believe that a significant dose reduction can even reduce the early parotid gland dysfunction. Moreover, based on the ‘as low as reasonably achievable’ principle, the technique should be practiced as it can reduce the parotid gland dose without disproportionate constraints or sacrifices.

In the RTOG 0933 trial, the D_100%_ of the hippocampus exceeding 10 Gy and maximal hippocampal dose exceeding 17 Gy were considered unacceptable deviations and required replanning before treatment initiation [4]. In this study, regardless of the presence of the parotid gland dose reduction, the D_100%_ of the hippocampus exceeded 10 Gy in approximately 50% cases of the 2.5 cm dynamic jaw (data not shown). However, the D_100%_ did not exceed 10 Gy in any cases of the 1.0 cm fixed jaw. Rong *et al.* predicted that the larger jaw size contributes to a wider dose penumbra, which would compromise hippocampus sparing [14]. The results of this study support the prediction of Rong *et al*. Thus, increasing the priority to the D_100%_ of the hippocampus is a method to satisfy its dose constraint during the computation of dose optimization. However, the method would be difficult to meet the constraint to the D_98%_ of the PTV_eval_, which competes with the D_100%_ of the hippocampus. As shown in Table 2, the D_98%_ of the PTV_eval_ was also close to its dose constraint (D_98%_ ˃ 25 Gy). As an alternative solution and a potential effective method, the use of a head tilt device can be considered, which is expected to reduce the D_100%_ of the hippocampus and to increase the D_98%_ of the PTV_eval_ [21]. Conversely, the D_100%_ of the hippocampus could be suppressed to ≤11.5 Gy in all cases using the 2.5 cm dynamic jaw. The relationship between the slight dose increase from 10 Gy and the clinical effect, namely, the cognitive deterioration, remains unclear.

The delivery time in addition to dose distribution are important to mention. In fact, Rong *et al.* reported that the delivery time of HA-WBRT was 18 min [14]. Miura *et al.* reported that the delivery time of HA-WBRT with and without the head tilt was 1328 ± 89 s and 1517 ± 86 s, respectively [21]. The authors used only the 1.0 cm fixed jaw. In our study, the delivery time with the 1.0 cm fixed jaw was approximately 14 min, which is the shortest delivery time compared with previous reports. Table 3 provides a summary of the treatment planning parameters and delivery time, which is important to discuss the reason of the mentioned difference. Indeed, it has reported that the delivery time increases by using a smaller pitch [22] and by a larger modulation factor [23, 24]. According to Table 3, the pitch used by Rong *et al.* [14] and Miura *et al*. [21] was smaller than that used in this study. Additionally, Miura *et al.* [21] used a larger modulation factor than that used in this study. Therefore, this study allowed shorter delivery time than the results of previous studies owing to the adoption of a larger pitch and a smaller modulation factor set in the treatment planning. Interestingly, as shown in Tables 2 and 3, the delivery time of the 2.5 cm dynamic jaw was shorter (approximately 60% reduction) than that of the 1.0 cm fixed jaw. The shortening of the delivery time is extremely promising and may allow to reduce the restraint due to the patient fixation device and the patient movement during treatment. The use of the 2.5 cm dynamic jaw would be a selectable undertaking account of the delivery time if the reduction of the D_100%_ of the hippocampus and maintenance of the D_98%_ of the PTV_eval_ may be achieved.

**Table 3:** Planning parameters

	Jaw width and delivery mode	Pitch	Modulation factor	Delivery time
Rong *et al.* [14]	1.0 cm fixed	0.215	2.0	18 min
Miura *et al.* [21]	1.0 cm fixed	0. 20	3.0	22 min (1328 s)^*^
				25 min (1517 s)
This study	1.0 cm fixed	0.43	2.0	14 min (836 s), 14 min (854 s)^*^^*^
	2.5 cm dynamic	0.43	2.0	6 min (363 s), 6 min (375 s)^*^^*^

^*^with the head tilt

^*^^*^with the parotid gland dose reduction

This study has some limitations. First, the dose optimization parameters stated in Table 1 were standardized for all patients, with 200 iterations to ease comparison of plans. Therefore, each patient’s treatment plan might further improve the quality of the dose distribution. Moreover, the analytical number of cases used in this study was small. The use of more cases will allow to improve the statistical accuracy of the obtained results. Finally, the relationship between the salivary function and a dose reduction in the parotid gland with the DB mode is unclear. Therefore, further clinical investigations on this topic are warranted. This study represents a pioneering development on the decrease of side effects in WBRT patients and could be implemented in future clinical trials.

## CONCLUSION

This study aimed to reduce the dose to the parotid gland in addition to sparing the hippocampus in WBRT using HT. By adding the parotid gland on the DB mode to the dose constraint, the dose to the parotid gland was reduced by approximately 70% without extending the delivery time. There was no significant deterioration in the dose distribution when the parotid gland dose in the HA-WBRT plan was reduced. In addition, compared with the plan employing the 1.0 cm fixed jaw, the plan employing the 2.5 cm dynamic jaw was more practical when it is possible to reduce the D_100%_ of the hippocampus and maintain the D_98%_ of the PTV_eval_ because the delivery time was reduced by approximately 60%.

## CONFLICT OF INTEREST

The aurhors declare they have no conflicts of interest.

## AUTHOR CONTRIBUTION STATEMENT

Hidetoshi Shimizu devised the study, performed analysis and interpretation of data and drafted the manuscript, supported by Koji Sasaki. Koji Sasaki, Takahiro Aoyama, Hiroyuki Tachibana, Yutaro Koide, Tohru Iwata, Tomoki Kitagawa and Takeshi Kodaira were involved in the study design and contributed significantly to the editing of the manuscript. All authors read and approved the final manuscript.

## APPENDIX

(1)
\begin{align*} {\rm HI}= \frac{D_{2\%}-D_{98\%}}{D_{50\%}} \times 100[\%] \end{align*}

(2) \begin{align*} {\rm CI} = \frac{TV_{RI}}{TV} \times \frac{TV_{RI}}{V_{RI}} \end{align*}

In equation (1), D_2%_, D_98_ and D_50%_ indicate the doses of 2%, 98% and 50% of the PTV_eval_, respectively. In equation (2), *TV* is the PTV_eval_, *TV*_RI_ is the PTV_eval_ covered by the reference isodose line, and *V*_RI_ is the volume inside the reference isodose line. In this study, the reference isodose was 30 Gy.
